# Prevalence of crisis pregnancy center attendance among women in four U.S. states

**DOI:** 10.1371/journal.pone.0324228

**Published:** 2025-06-04

**Authors:** Teresa J.K. Yang, Mikaela H. Smith, Megan L. Kavanaugh, JaNelle M. Ricks, Maria F. Gallo

**Affiliations:** 1 College of Medicine, The Ohio State University (OSU), Columbus, Ohio, United States of America; 2 College of Public Health, OSU, Columbus, Ohio, United States of America; 3 Guttmacher Institute, New York, New York, United States of America; SUNY Downstate Health Sciences University, UNITED STATES OF AMERICA

## Abstract

**Objectives:**

Crisis pregnancy centers (CPCs) typically hold missions of preventing abortion, opposing contraception, and promoting abstinence outside of marriage. They often lack transparency about their services, posing as medical facilities or even as abortion clinics. Given the lack of evidence on the extent to which people use crisis pregnancy centers, we sought to quantify the prevalence of ever attendance at a CPC among adult, reproductive-aged women from Survey of Women data from four states.

**Study design:**

We analyzed cross-sectional data from population-representative surveys conducted among adult, reproductive-age women in 2018–2019 in Iowa (N = 2,425) and in 2019–2020 in Arizona (N = 2,132), New Jersey (N = 2,132), and Wisconsin (N = 2,095). Using survey weights, we calculated the prevalences of ever CPC attendance among those with a history of pregnancy or testing for pregnancy. We focused on this subset as this comprises the women who might have had cause to attend a CPC. We also used Poisson regression to test associations between demographic correlates and ever attendance by state.

**Results:**

Prevalence of ever CPC attendance in adult, reproductive-age women with a history of pregnancy or testing for pregnancy was statistically significantly higher in Arizona (20.2%; 95% CI, 17.6%-23.1%) compared to Iowa (14.5%; 95% CI, 12.6%-16.7%), Wisconsin (14.3%; 95% CI, 12.1%-16.8%).), and New Jersey (11.6%; 95% CI, 9.6%-13.8%). Age, race/ethnicity, and socioeconomic status were not correlated with ever CPC attendance among women with a history of pregnancy or testing for pregnancy in Arizona, Iowa, and New Jersey. In Wisconsin, prevalence was lower among those in the lowest socioeconomic stratum.

**Conclusions:**

Ever attendance at CPCs is not rare, ranging from 11.6%-20.2% in the four states evaluated. The present study serves as an important baseline given that the prevalence may change as pregnancy options become increasingly restricted.

## Introduction

Access to reproductive healthcare is a public health imperative. Under the principles of reproductive justice, people have the right to 1) have a child and dictate the conditions under which they give birth, 2) not have a child and have access to complete, unbiased, medically accurate information when making pregnancy-related decisions, and 3) parent the children that they already have in safe and healthy communities with essential social support [1]. The ability to uphold these tenets is increasingly jeopardized as access to comprehensive, safe, accurate, and affordable reproductive healthcare is restricted [2].

Crisis pregnancy centers (CPCs) (sometimes referred to with other terms such as *pregnancy resource centers, pregnancy care centers* or *pregnancy centers*), are facilities that offer pregnancy tests, diapers, baby clothes, parenting resources, and pregnancy counselling [3]. Although they typically are not licensed medical clinics, many offer limited ultrasound examination [4,5]. Most CPCs are affiliated with religious organizations that aim to prevent abortion, oppose contraception, and promote abstinence until marriage [6,7]. While CPC staff are drawn to the mission of dissuading people from having an abortion, a review of their service data found that their work often centers on helping people with material resources for their pregnancy and only rarely results in influencing other’s views about abortion [3].

CPCs often lack transparency about their services, posing as medical facilities or even as abortion clinics [5,7–10]. Consequently, some people have difficulty in correctly identifying CPCs [11,12]. Moreover, CPCs frequently appear in internet searches for abortion, adding to patient confusion [13]. This lack of transparency may introduce delays in receiving either abortion or prenatal care [11,14].

CPCs pose a public health risk by providing misleading, manipulative, or inaccurate health information [15]. The Society for Adolescent Health and Medicine and the North American Society for Pediatric Gynecology warn of the risks from CPCs failing to comply with sexual and reproductive health standards [16]. Medical inaccuracies listed on CPC websites or stated during CPC counselling include citing spurious links between abortion and breast cancer, poor mental health, so-called “post-abortion stress,” and infertility, and making inaccurate claims about condom ineffectiveness [5–7,17]. Furthermore, CPC staff use stigmatizing language to try to coerce people [18] and make “inflammatory” statements regarding abortion [10].

In 2018, more than 2,500 CPC facilities were operating in the U.S., three times more than the number of abortion facilities [4,19]. Despite their pervasiveness and potential for negative health consequences, the use of CPCs remains largely understudied. The present study draws on data from four states – Arizona, Iowa, New Jersey, and Wisconsin – originally identified under a larger multi-year initiative to track the impacts of policy changes on publicly funded family planning healthcare systems and their patients [20]. In 2017, access to abortion across these states varied widely: about 20% of Arizona counties, 7% of Iowa counties, 67% of New Jersey counties, and 3% of Wisconsin counties had an abortion provider [21]. However, states are not isolated units, and women cross state lines to receive abortion care. Prior to Dobbs in 2022, women drove an average of 16.7 miles in Arizona, 41.3 miles in Iowa, 6.2 miles in New Jersey, and 44.7 miles in Wisconsin to receive abortion care [22]. In contrast, 80% of Arizona counties, 28% of Iowa counties, 95% of New Jersey counties, and 51% of Wisconsin counties had a CPC in 2018 [19]. In 2018, Arizona had 3.5 CPCs per 100,000 reproductive-age women; Iowa had 7.2 per 100,000; New Jersey had 1.8 per 100,000; and Wisconsin had 4.3 per 100,000 [4]. We sought to quantify the prevalence of women who have ever attended a CPC in each of these four states and to identify factors associated with ever attendance.

## Materials and methods

### Data

The Surveys of Women are a set of population-representative surveys on reproductive knowledge, attitudes, and behaviors of women aged 18–44 years conducted in several states [23]. We analyzed data from the first wave of surveys in four states: Arizona (October 2019 – July 2020), Iowa (September 2018 – June 2019), New Jersey (October 2019 – July 2020), and Wisconsin (October 2019 – July 2020). We conducted the analyses in June to October 2024. Eligibility was restricted to those who selected a response other than “male” in the eligibility screener and those who provided written consent. The institutional review board (IRB) at NORC at the University of Chicago provided ethical review of the survey, and the Ohio State University IRB deemed the present analysis to be exempt from further review. The authors did not have access to information at any time that could identify individual participants.

The survey methods have been described elsewhere [23]. In brief, randomly-selected households were mailed letters requesting that they complete the survey online and nonrespondents were mailed a paper survey to complete. The response rate for the initial survey wave was 32% in Arizona (N = 2055), 38% in Iowa (N = 2384), 24% in New Jersey (N = 2172), and 38% in Wisconsin (N = 2041). For all percentages and regression modeling, we used NORC’s survey weights to adjust for the probabilities of selection, nonresponse and post-stratification imputation and raking.

### Measures

We assessed ever CPC attendance based on the question, “Have you ever visited a pregnancy resource center, also known as a crisis pregnancy center, for pregnancy-related care?” Response options consisted of “yes,” “no,” “not sure,” and “prefer not to answer.” For descriptive analyses, we treated CPC attendance as a three-category variable; those selecting “prefer not to answer” (N = 110) were coded as missing. For inferential analyses, we created a binary variable comparing those who selected “yes” to all others.

We analyzed our outcome by two groups: women overall (results reported in supplemental files only) and those who had ever been pregnant (i.e., reporting ever had unplanned pregnancy, ever had an abortion, ever had live birth, or ever had a miscarriage; N = 5266) or who had ever taken a pregnancy test (N = 6677). We chose to subset analyses based on history of pregnancy or testing for pregnancy, given that these populations would be more likely to have had reason to seek services at a CPC. We excluded respondents (N = 1789) from our sub-group analysis if they were missing on either pregnancy history or testing unless they had non-missing data for either and their response indicated that they had either been pregnant or had tested for pregnancy.

We assessed three demographic factors as possible correlates of ever CPC attendance: age and two factors – race/ethnicity and socioeconomic status (SES) – that were previously identified as correlates of CPC attendance in Ohio [24]. We included age as a categorical variable, with options of 18–29, 30–39, and 40–44 years. We use three different categorizations for race/ethnicity given the demographic differences between states and the categories provided by NORC. For Arizona, we grouped respondents into Hispanic/Latina, non-Hispanic White (referent), and non-Hispanic another race (which included Black, Asian, American Indian/Alaskan Native, Hawaiian/Pacific Islander, and multiracial). For Iowa and Wisconsin, we grouped respondents into non-Hispanic White (referent) versus another race. For New Jersey, we grouped respondents into non-Hispanic Black, non-Hispanic White (referent), non-Hispanic another race, and Hispanic/Latina. For SES, we combined two variables provided by NORC that captured respondents’ household income and education level completed. We created a four-category variable that indicated whether respondence had a household income below $75,000 and no college degree (referent), income of $75,000 or more without a college degree, income below $75,000 with a college degree, and income of $75,000 or more with a college degree. NORC used hot-deck imputation to account for the records with missing values for these demographic variables. Six respondents, all from Iowa, were missing imputed values on these variables and so we excluded them from the analysis.

### Analysis

For each state, we first calculated the prevalence and 95% confidence intervals (CIs) of ever attending a CPC for two groups: overall, and among those who reported a history of pregnancy or testing for pregnancy. Second, for each state separately among those with a history of pregnancy or testing for pregnancy, we calculated unadjusted and adjusted prevalence ratios (PRs) and 95% CIs of ever attending a CPC based on the three demographic factors (age, race/ethnicity, SES) using Poisson regression models with robust standard errors. Individual states used different categories of race/ethnicity in their survey to reflect their population. The adjustment set included the three factors.

## Results

In Arizona, 42% of the women were Hispanic/Latina and 47% were non-Hispanic White; 45% were in the lowest SES group (Table 1). In Iowa, 84% of women were White, and 41% had low income and education. In New Jersey, 49% were non-Hispanic White and 21% were Hispanic/Latina; 27% were in the lowest SES stratum. In Wisconsin, 77% were non-Hispanic White and 38% had low education and income.

**Table 1:** Demographic characteristics by state^a^ among women 18-44 years of age with history of pregnancy or testing for pregnancy.

	Arizona		Iowa		New Jersey		Wisconsin	
	No.^b^	(%)^c^	No.^b^	(%)^c^	No.^b^	(%)^c^	No.^b^	(%)^c^
Age in years								
18-29	363	(39)	474	(37)	368	(32)	375	(34)
30-39	813	(42)	893	(42)	783	(45)	770	(44)
40-44	559	(19)	566	(21)	559	(23)	545	(21)
Race and ethnicity								
Non-Hispanic Black	50	(4)	35	(4)	204	(15)	94	(9)
Hispanic/Latina	539	(43)	81	(3)	325	(23)	97	(7)
Non-Hispanic multiracial or another	124	(7)	123	(9)	173	(14)	74	(8)
Non-Hispanic White	1022	(45)	1694	(84)	1008	(48)	1425	(76)
Socioeconomic status								
Some college or less, < $75K	594	(46)	664	(41)	380	(27)	537	(37)
Some college or less, < $75K	273	(28)	293	(25)	235	(26)	287	(27)
Bachelor’s degree or higher, < $75K	313	(8)	363	(10)	280	(8)	299	(10)
Bachelor’s degree or higher, ≥ $75K	555	(18)	613	(24)	815	(39)	567	(26)
Ever attended a CPC								
Yes	323	(20)	300	(15)	188	(12)	237	(14)
No	1379	(78)	1588	(83)	1470	(85)	1414	(83)
Unsure	33	(2)	45	(2)	52	(3)	39	(3)

^a^Survey conducted in 2018–2019 in Iowa and in 2019–2020 in Wisconsin, Arizona, and New Jersey; ^b^Unweighted number; ^c^Weighted percent.

CPC, crisis pregnancy center.

**Fig 1:**
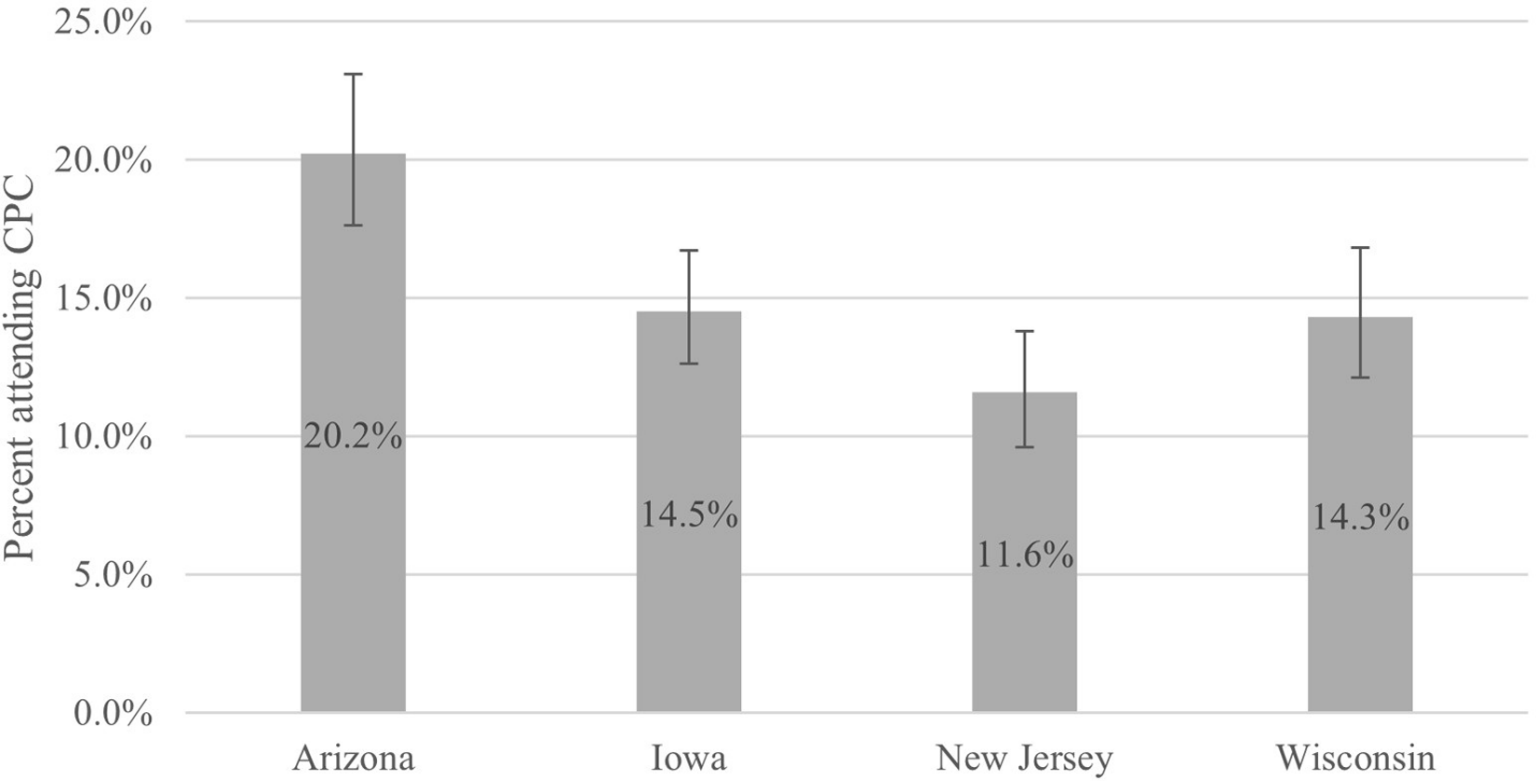
Percentage (weighted) of ever attendance at crisis pregnancy center by state for women aged 18-44 years with 95% confidence intervals among those with history of pregnancy or testing for pregnancy.

Prevalence of ever CPC attendance varied by state (Fig 1). Among women with a history of pregnancy or testing for pregnancy, Arizona women also had a statistically significantly higher prevalence of attending a CPC (20.2%; 95% CI, 17.6%-23.1%) relative to those in Wisconsin (14.3%; 95% CI, 12.1%-16.8%), Iowa (14.5%; 95% CI, 12.6%-16.7%), and New Jersey (11.6%; 95% CI, 9.6%-13.8%) The prevalence of CPC attendance also was higher in Arizona compared to women in other states when assessed among women overall (i.e., not restricted to those with a history of pregnancy or testing for pregnancy) (S1 Table and S1 Fig).

Among women with a history of pregnancy or testing for pregnancy, the demographic factors assessed (age, race/ethnicity, and SES) were not associated with prevalence of CPC attendance in the unadjusted (Table 2) or adjusted analyses (Table 3) for the four states with one exception. In Wisconsin, among those with a history of pregnancy or testing for pregnancy, those in the highest SES stratum had a statistically significantly higher prevalence of CPC attendance (PR, 1.1; 95% CI, 1.1–1.2) compared to those in the lowest in both the unadjusted and adjusted analyses.

**Table 2:** Unadjusted correlates of ever attendance at crisis pregnancy center among women with a history of pregnancy or testing for pregnancy.

	Arizona		Iowa		New Jersey		Wisconsin	
	PR	(95% CI)	PR	(95% CI)	PR	(95% CI)	PR	(95% CI)
Age in years								
18-29	ref.		ref.		ref.		ref.	
30-39	0.9	(1.0-1.0)	0.9	(0.9-1.0)	1.0	(0.9-1.1)	0.9	(0.9-1.0)
40-44	0.9	(0.8-1.0)	0.9	(0.9-1.0)	0.9	(0.9-1.0)	1.0	(0.9-1.0)
Race/ethnicity (Arizona)								
Hispanic/Latina	0.9	(0.8-1.0)						
Non-Hispanic White	ref.							
Non-Hispanic multiracial or another race	0.9	(0.8-1.0)						
Race/ethnicity (Iowa, Wisconsin)								
Non-Hispanic White				ref.			ref.	
Hispanic, or multiracial or another race			0.9	(0.8-1.0)			0.9	(0.8-1.0)
Race/ethnicity (New Jersey)								
Non-Hispanic Black					1.0	(0.9-1.1)		
Hispanic/Latina					1.0	(0.9-1.1)		
Non-Hispanic White					ref.			
Non-Hispanic multiracial or another race					1.1	(1.0-1.1)		
Socioeconomic status								
Some college or less, < $75K	ref.		ref.		ref.		ref.	
Some college or less, < $75K	1.0	(0.9-1.1)	1.0	(0.9-1.1)	1.0	(0.9-1.0)	1.1	(1.0-1.2)
Bachelor’s degree or higher, < $75K	1.1	(1.0-1.2)	1.0	(1.0-1.1)	1.0	(0.9-1.1)	1.1	(1.0-1.2)
Bachelor’s degree or higher, ≥ $75K	1.1	(1.0-1.2)	1.0	(1.0-1.1)	1.1	(1.0-1.1)	1.1	(1.1-1.2)

CI, confidence interval; PR, prevalence ratio.

**Table 3:** Adjusted correlates of ever attendance at crisis pregnancy center among women with a history of pregnancy or testing for pregnancy.

	Arizona		Iowa		New Jersey		Wisconsin	
	PR	(95% CI)	PR	(95% CI)	PR	(95% CI)	PR	(95% CI)
Age in years								
18-29	ref.		ref.		ref.		ref.	
30-39	0.9	(1.0-1.0)	0.9	(0.9-1.0)	1.0	(0.9-1.0)	0.9	(0.8-1.0)
40-44	0.9	(0.8-1.0)	0.9	(0.9-1.0)	0.9	(0.9-1.0)	0.9	(0.9-1.0)
Race/ethnicity (Arizona)								
Hispanic/Latina	0.9	(0.8-1.0)						
Non-Hispanic White	ref.							
Non-Hispanic multiracial or another race	0.9	(0.8-1.0)						
Race/ethnicity (Iowa, Wisconsin)								
Non-Hispanic White				ref.			ref.	
Hispanic, or multiracial or another race			0.9	(0.8-1.0)			0.9	(0.8-1.0)
Race/ethnicity (New Jersey)								
Non-Hispanic Black					1.0	(0.9-1.1)		
Hispanic/Latina					1.0	(0.9-1.1)		
Non-Hispanic White					ref.			
Non-Hispanic multiracial or another race					1.1	(1.0-1.1)		
Socioeconomic status								
Some college or less, < $75K	ref.		ref.		ref.		ref.	
Some college or less, < $75K	1.0	(0.9-1.1)	1.0	(0.9-1.1)	1.0	(0.9-1.0)	1.1	(1.0-1.2)
Bachelor’s degree or higher, < $75K	1.1	(1.0-1.2)	1.0	(1.0-1.1)	1.0	(0.9-1.1)	1.1	(1.0-1.2)
Bachelor’s degree or higher, ≥ $75K	1.1	(1.0-1.2)	1.0	(1.0-1.1)	1.1	(1.0-1.1)	1.1	(1.1-1.2)

CI, confidence interval; PR, prevalence ratio.

## Discussion

Population-based surveys of adult, reproductive-aged women revealed the lifetime prevalence of ever attending a CPC among women with a history of pregnancy or pregnancy testing to be 20.2% in Arizona, 14.3% in Wisconsin, 11.6% in New Jersey in 2019–2020 and 14.5% in Iowa in 2018–2019. To our knowledge, only one other study has examined prevalence of CPC attendance: a population-representative survey in Ohio found prevalence of ever CPC attendance among adult, reproductive-age women in the state to be 13.5% in 2018–2019 [24]. The estimated prevalence of CPC attendance measured in Ohio is within the range of prevalence estimates for adult, reproductive-age women measured in the present report of four states. Two additional studies used convenience samples to document CPC attendance. A study in Louisiana, which surveyed patients from 1 abortion center (n = 114) and 3 prenatal clinics (n = 269), showed that only 7 of the abortion patients and 14 of the prenatal patients had visited a CPC for their current pregnancy [14]. A Google Ads Abortion Access study using data from pregnant women searching online for an abortion found that 13.1% of women had visited a CPC during their current pregnancy [11]. However, because these two studies relied on convenience sampling, their target populations are unknown.

SES status was not correlated to CPC utilization among women with a history of pregnancy or testing for pregnancy in Arizona, Iowa, and New Jersey. However, in Wisconsin, those in the lowest SES stratum had lower prevalence of ever attending a CPC compared to those in the highest SES stratum, suggesting the involvement of other structural factors such as CPC location or the role of a baseline level of affluence that is needed to access CPC services in Wisconsin. This finding contrasts with the population-representative survey of Ohio women, which found that women in the lowest SES category (defined by education and income) had higher prevalence of ever attending a CPC [24].

A strength of the present study was the use of a population-representative sample of adult, reproductive-age women in the four states. The study design allows us to calculate prevalence and generalize findings for these populations. A primary limitation was the use of self-reported data, which could have resulted in misclassification – in either direction – of ever CPC attendance. For example, some women might have faced more stigma in seeking abortion or care for an unintended pregnancy and thus might have been less likely to report CPC attendance. This could have biased the prevalence estimates downward and could have interfered with our assessment of correlates of ever CPC attendance. This study also analyzed states as separate units, though in practice women may cross state lines to receive pregnancy or abortion care. Furthermore, studies have demonstrated that some women, especially those with low health literacy, are unaware that locations are CPCs [11,12]. Women might fail to recognize that a location is a CPC or they might mistakenly infer that health center is a CPC. Importantly, this analysis examined individual characteristics associated with CPC attendance but did not account for broader, structural factors that impact this outcome. We did not examine the role of broader structural factors (e.g., religion, systematic disadvantage, and health insurance) that may affect CPC attendance. We excluded from our analysis women who reported being unsure (n = 187) of whether they had attended a CPC. The four-level measure for socioeconomic status might have failed to capture important differences between individuals and states. Finally, the survey was administered to only women at least 18 years of age and therefore we cannot comment on CPC utilization among minors.

Women’s use of CPCs might change following the U.S. Supreme Court’s decision in *Dobbs v. Jackson* in 2022 to move the decision-making about access to abortion care to the states. As reproductive health becomes more heavily surveilled, women may attend CPCs less often due to privacy concerns. Because CPCs are not licensed medical facilities, they are not required to protect their clients’ privacy, and some women might become reluctant to disclose their pregnant status. Conversely, CPC attendance could rise as abortion care becomes increasingly restricted or banned in their state. Depending on their state of residence, pregnant people might face pressure to confirm their pregnancy more quickly if they have a smaller window to obtain a legal abortion in their state or if they need to travel out of state to receive care. Thus, some may seek a rapid pregnancy confirmation via a CPC. Greater attendance at CPCs could have negative consequences as more people would be exposed to misinformation and stigma. Our study, drawing on data from the period prior to these shifts in the reproductive landscape, serves as an important baseline for understanding the role that CPCs play in the broader healthcare landscape for pregnant people going forward; future studies should continue to monitor CPC attendance in the wake of greater restrictions on abortion access and reproductive healthcare more generally.

## Supporting information

S1 Table Demographic characteristics by state^a^ among women 18–44 years of age. (DOCX)

S1 Fig Prevalence of CPC attendance by state among women aged 18–44 years, overall. (JPG)
