# Supplementary material for: Prevalence of crisis pregnancy center attendance among women in four U.S. states
Source: PLoS One. 2025 Jun 4;20(6):e0324228. doi: 10.1371/journal.pone.0324228 (PMC12136328; doi:10.1371/journal.pone.0324228)
Supplement: S1 Table — (DOCX) [file pone.0324228.s001.docx]

**Supplemental Table 1. Demographic characteristics by state^a^ among women 18-44 years of age.**

|  | **Arizona** | | **Iowa** | | **New Jersey** | | **Wisconsin** | |
| --- | --- | --- | --- | --- | --- | --- | --- | --- |
|  | No.^b^ | (%)^c^ | No.^b^ | (%)^c^ | No.^b^ | (%)^c^ | No.^b^ | (%)^c^ |
| Age in years |  |  |  |  |  |  |  |  |
| 18-29 | 539 | (46) | 742 | (45) | 639 | (42) | 584 | (43) |
| 30-39 | 889 | (37) | 985 | (37) | 883 | (39) | 830 | (38) |
| 40-44 | 594 | (17) | 601 | (18) | 591 | (19) | 583 | (18) |
| Race and ethnicity |  |  |  |  |  |  |  |  |
| Non-Hispanic Black | 56 | (4) | 40 | (3) | 243 | (14) | 103 | (8) |
| Hispanic/Latina | 628 | (42) | 100 | (3) | 387 | (21) | 120 | (7) |
| Non-Hispanic multiracial or another | 152 | (7) | 159 | (10) | 236 | (16) | 89 | (7) |
| Non-Hispanic White | 1186 | (47) | 2029 | (84) | 1247 | (49) | 1685 | (77) |
| Socioeconomic status |  |  |  |  |  |  |  |  |
| Some college or less, <$75K | 697 | (45) | 803 | (41) | 466 | (27) | 651 | (38) |
| Some college or less, <$75K | 311 | (28) | 336 | (24) | 291 | (27) | 328 | (27) |
| Bachelor's degree or higher, <$75K | 395 | (9) | 492 | (12) | 385 | (9) | 388 | (11) |
| Bachelor's degree or higher, ≥$75K | 619 | (18) | 697 | (23) | 971 | (37) | 630 | (24) |
| Ever attended a CPC |  |  |  |  |  |  |  |  |
| Yes | 329 | (17) | 310 | (12) | 191 | (9) | 239 | (12) |
| No | 1657 | (81) | 1969 | (86) | 1861 | (88) | 1717 | (86) |
| Unsure | 36 | (2) | 49 | (2) | 61 | (3) | 41 | (3) |
| ^a^Survey conducted in 2018-2019 in Iowa and in 2019-2020 in Wisconsin, Arizona, and New Jersey; ^b^Unweighted number; ^c^Weighted percent  CPC = crisis pregnancy center | | | | | | | | |
