# Supplementary figures and images for: Prevalence of crisis pregnancy center attendance among women in four U.S. states
Source: PLoS One. 2025 Jun 4;20(6):e0324228. doi: 10.1371/journal.pone.0324228 (PMC12136328; doi:10.1371/journal.pone.0324228)

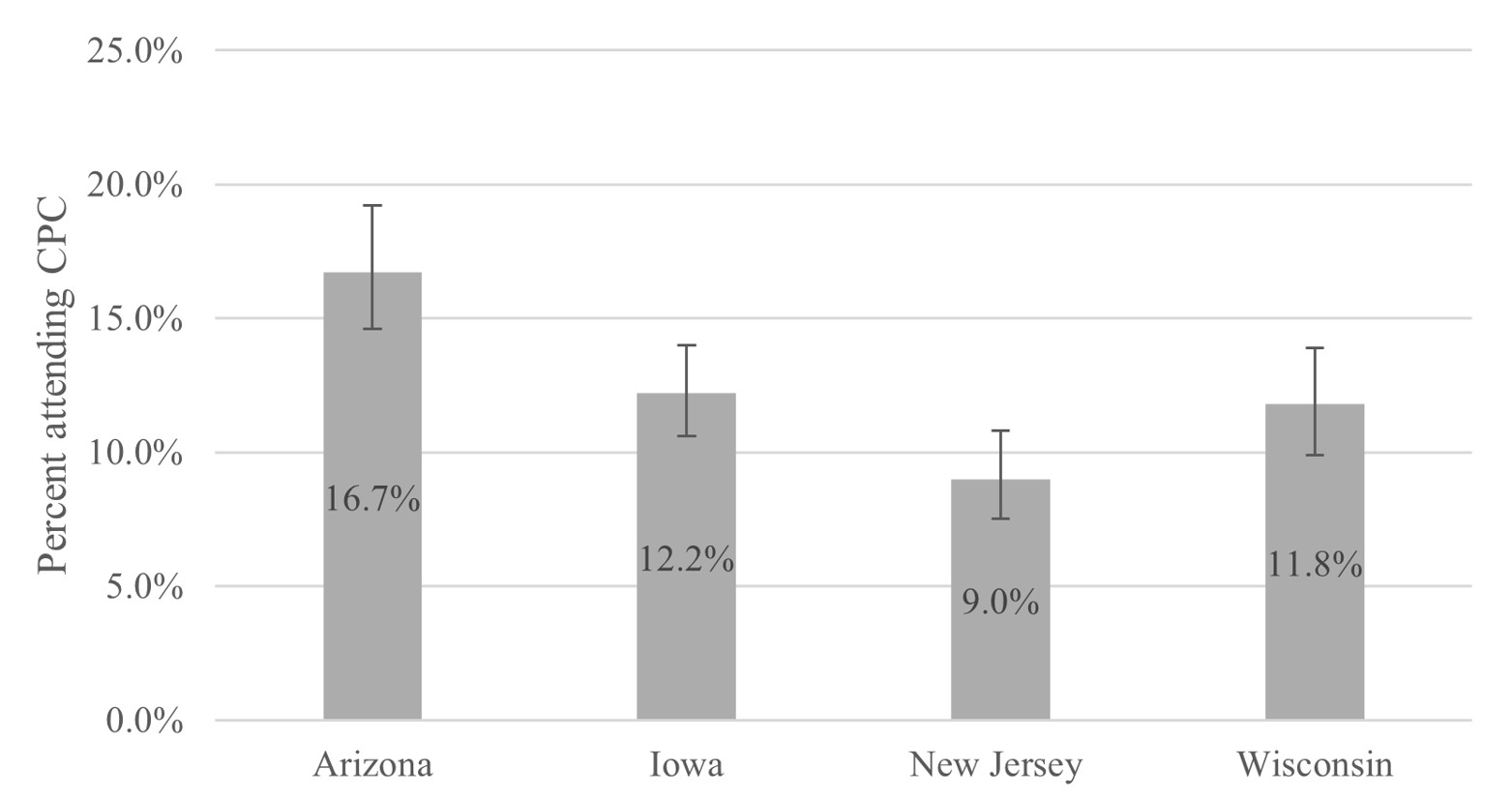

Supplement: S1 Fig — (JPG) [file pone.0324228.s002.jpg]
